# Local *V*_OC_ Measurements by Kelvin Probe Force Microscopy Applied on P-I-N Radial Junction Si Nanowires

**DOI:** 10.1186/s11671-019-3230-5

**Published:** 2019-12-30

**Authors:** Clément Marchat, Letian Dai, José Alvarez, Sylvain Le Gall, Jean-Paul Kleider, Soumyadeep Misra, Pere Roca i Cabarrocas

**Affiliations:** 1 Institut Photovoltaïque d’Ile-de-France (IPVF), 18 Boulevard Thomas Gobert, 91120 Palaiseau, France; 2 Génie électrique et électronique de Paris (GeePs), UMR CNRS 8507, CentraleSupélec, Univ. Paris-Sud, Université Paris-Saclay, Sorbonne Universités, UPMC Univ Paris 06, 11 rue Joliot Curie, 91192 Gif-sur-Yvette, France; 3 Laboratoire de Physique et Interfaces et des Couches Minces (LPICM), UMR CNRS 7647, CNRS, Ecole Polytechnique, Université Paris-Saclay, 91128 Palaiseau, France; 4 Laboratoire de Physique de la Matière Condensée (LPMC), UMR CNRS 7643, École Polytechnique, 91128 Palaiseau, France

**Keywords:** Solar cells, KP, Nanoscale, Characterization, Surface photovoltage spectroscopy, SPS, Ideality factor, Band bending

## Abstract

This work focuses on the extraction of the open circuit voltage (*V*_OC_) on photovoltaic nanowires by surface photovoltage (SPV) based on Kelvin probe force microscopy (KPFM) measurements. In a first approach, P-I-N radial junction (RJ) silicon nanowire (SiNW) devices were investigated under illumination by KPFM and current-voltage (I-V) analysis. Within 5%, the extracted SPV correlates well with the *V*_OC_. In a second approach, local SPV measurements were applied on single isolated radial junction SiNWs pointing out shadowing effects from the AFM tip that can strongly impact the SPV assessment. Several strategies in terms of AFM tip shape and illumination orientation have been put in place to minimize this effect. Local SPV measurements on isolated radial junction SiNWs increase logarithmically with the illumination power and demonstrate a linear behavior with the *V*_OC_. The results show notably that contactless measurements of the *V*_OC_ become feasible at the scale of single photovoltaic SiNW devices.

## Introduction

Semiconductor nanostructures attract a great deal of research interest because of their nanoscale properties that offer a great potential for improving performances in existing devices. Nanowire arrays based on radial junctions (RJs) are promising nanostructures for photovoltaic (PV) applications due to their light trapping and carrier collection properties [1, 2] that are purposely combined for enhancing solar efficiency with respect to conventional planar structures. The efficiency of nanowire solar cells may be limited by damaged nanowire junctions in the array; nevertheless, efficiencies up to 9.6 % have been already demonstrated for silicon nanowire (SiNW) RJs based on Si thin-film technology [3]. The characterization of such structures remains a critical issue, and notably the possibility to characterize the photoelectrical performances of individual nanowires is an added value for the improvement of the final device.

In the present study, we used Kelvin probe force microscopy (KPFM) to evaluate the local open-circuit voltage (*V*_OC_) on SiNW RJs. The analysis of *V*_OC_ has been successfully evaluated by KPFM on several types of photovoltaic technologies, mostly planar structures [3, 4]. However, KPFM analysis on PV nanodevices is not straightforward notably due to that it can require to perform measurements in both dark and illumination conditions to extract the surface potential variation, named surface photovoltage (SPV).

Our first approach to probe the local *V*_OC_ of RJ SiNWs was to analyze completed devices. The term completed refers to RJ SiNW solar cells that are finalized with ITO as front electrode. The following completed devices were sequentially characterized by current-voltage (I-V) and KPFM measurements. Both measurements were performed under dark and illumination conditions with the final goal to extract and compare *V*_OC_ and SPV. Our second approach was to analyze single isolated RJ SiNWs that were not coated by ITO. We particularly aimed at optimizing the KPFM signal under illumination avoiding many artifacts that may result in the underestimation of the SPV value. Each single isolated RJ SiNW will be referenced as isolated device.

Finally to complete the results, macroscopic Kelvin probe technique was also applied on a completed RJ device and on a bunch of isolated devices. This was done under illumination at different wavelengths in order to perform surface photovoltage spectroscopy (SPS).

## Materials and Methods

### SiNW Growth and Radial P-I-N Junction Device Fabrication

The RJ SiNWs were prepared on a substrate of ZnO:Al coated Corning glass (Cg). The SiNWs growth was done at a substrate temperature of 500°C by Plasma Enhanced Chemical Vapor Deposition (PECVD) and was mediated using Sn nanoparticles as catalysts. The P-I-N RJ was obtained by depositing thin conformal layers of intrinsic (80 nm) and then n-type (10 nm) hydrogenated amorphous Si (a-Si:H) also by PECVD at 175°C on the p-type SiNW core. The completed devices were finalized with a conformal deposition of ITO forming circular top contacts of diameter 4 mm defined by a mask during sputter-deposition. The full details of the fabrication are explained elsewhere [1, 5–7].

### Kelvin-Probe and Surface Photovoltage

KPFM measurements can be performed using two different modes, amplitude modulation (AM) and frequency modulation (FM). Both modes allow one to obtain the same contact potential difference (CPD) property value existing between the tip and the surface of the sample. The AM mode was the one chosen in this study, the reason being its greater measurement stability in presence of significant height variations such as those seen at the edge of the sample nanowires.

KPFM and SPV measurements were performed using a scanning probe microscopy system from HORIBA/AIST-NT (TRIOS platform) that offers several advantages. Indeed, for this atomic force microscope (AFM) the laser beam-based deflection system (LBBDS) employs a laser wavelength at 1310 nm that minimizes the possible photoelectric interactions with the sample [8–10**]**. This will be emphasized here by comparing data acquired using this platform with that obtained using an AFM system that uses a 690 nm wavelength for the LBBDS.

The TRIOS platform is well suited to study photoelectrical properties of materials since it includes three microscope objectives allowing the illumination of the sample from different directions (top, side and bottom). SPV measurements at the micro/nano scale are here obtained by subtracting the CPD in the dark to the CPD under illumination. This kind of measurement has previously been used to perform V_OC_ measurements of photovoltaic devices [5, 11]. The illumination of the sample was accomplished using an OXXIUS stabilized laser diode of wavelength 488 nm with a variable power module.

Two kinds of conductive AFM tips were used for the applied scanning probe measurements, the ARROW-EFM and the ATEC-EFM. Both of them have a doped silicon cantilever and a PtIr coating. Their difference lies in their shape with a conventional tip shape for the ARROW and a tilted shape for the ATEC.

Finally, SPV measurements at the nanoscale were complemented with macroscopic Kelvin Probe measurements with the possibility of varying the illumination wavelength in order to perform SPS measurements, i.e. spectrally resolved SPV measurements. To this purpose an ASKP200250 Kelvin Probe setup from KPTechnology equipped with a 2 mm diameter steel tip was used. This setup includes an illumination from the side coupling a halogen lamp source to a monochromator that covers the wavelength range from 400 nm to 1000 nm. Note that this configuration does not allow to perform SPV measurements at constant flux and for this particular reason only qualitative observations can be made.

### Macroscopic I-V measurements combined to KPFM

**Fig. 1:**
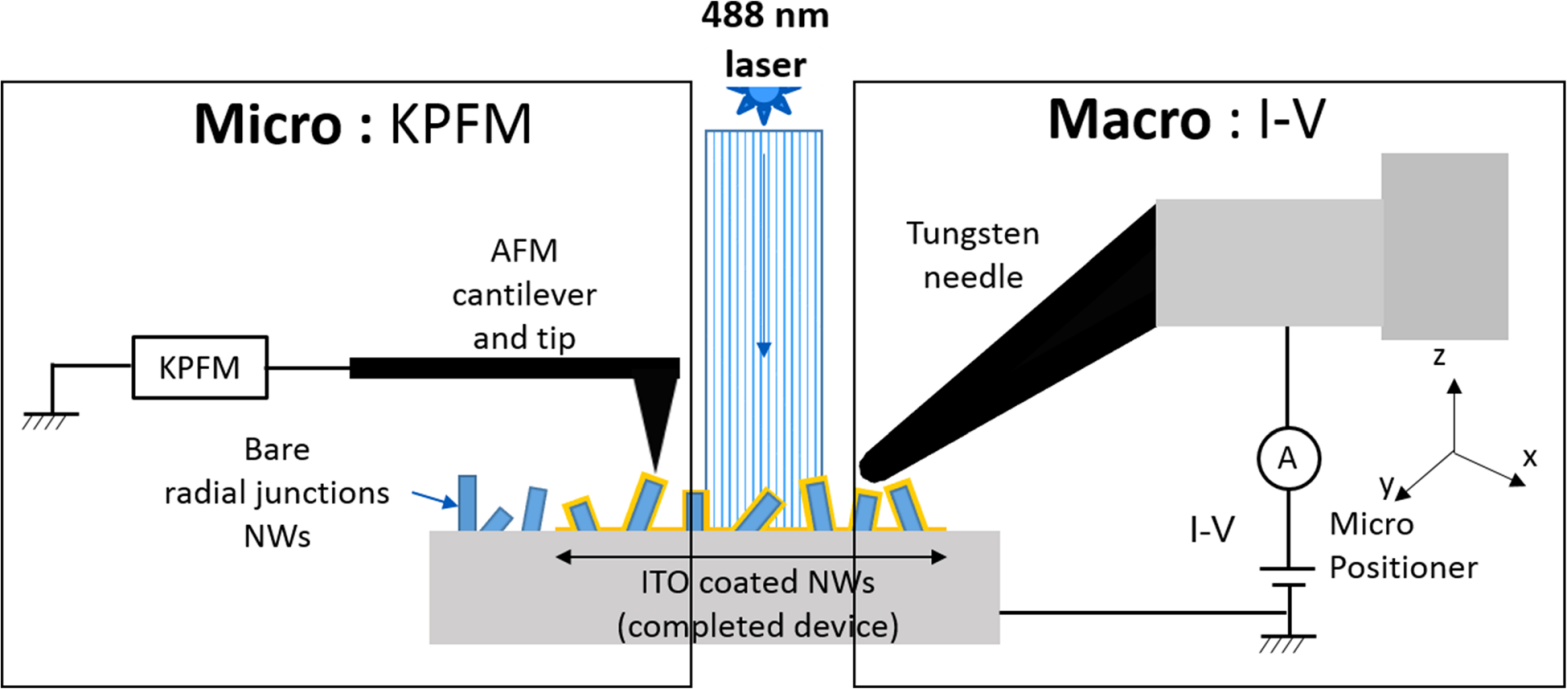
Schematics of the measurement setup for both KPFM and macroscopic I-V measurements

As previously indicated, our first approach was to perform macroscopic I-V measurements on a completed SiNW RJ device. To this purpose we used a KEITHLEY 2450 SourceMeter and a micro positioner with a tungsten needle that enables to contact the device while being under the AFM setup as schematized in Fig. 1.

I-V and KPFM measurements were performed under dark conditions and then using the same illumination described in the previous subsection, namely a laser source at 488 nm with adjustable power. The illumination was realized from the top side through a MITUTOYO 10X objective and the incident power illumination was calibrated in the range 70 -1000 μW.

KPFM measurements were further performed on the isolated devices with two kind of AFM tips, ARROW and ATEC. The illumination of the sample during the measurement was done from two directions, top and side, and using the same nominal power as the ones used previously on the completed device.

## Results and discussion

**Fig. 2:**
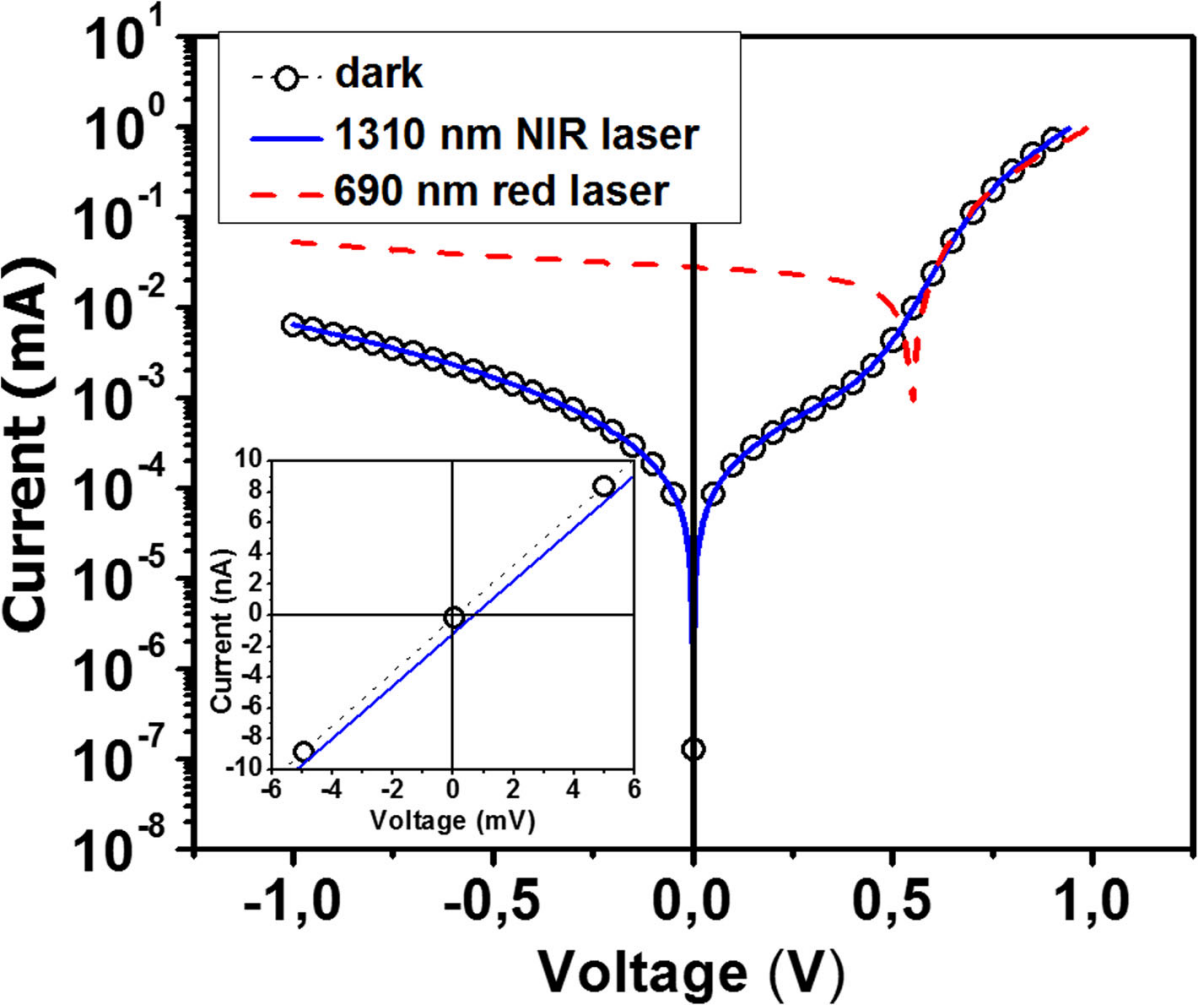
I-V curves obtained on a SiNW RJ device under dark conditions (black circles), with the 1310-nm laser beam of the TRIOS AFM (blue solid line) and with the 690-nm laser beam of the Enviroscope AFM (red dashed line). The principal graph illustrates the log |I|-V curves in the range − 1 V and + 1 V, and the insert graph represents an enlargement of the linear I-V curves between − 5 mV and + 5 mV

Before starting I-V and KPFM measurements, the impact of the AFM’s LBBDS was investigated. Indeed, it has already been shown that the wavelength of the LBBDS can have a significant interaction with photovoltaic samples [8–10] and so may influence electrical properties measurements with the AFM. Figure 2 illustrates the macroscopic I‑V measurements of a *completed* SiNW RJ device performed under dark conditions (LBBDS switched off) and when the LBBDS is kept on. As previously mentioned, measurements were also performed in a different AFM setup using a wavelength of 690 nm instead of 1310 nm for the LBBDS. The I-V curves obtained under dark conditions and with the LBBDS at 1310 nm are almost identical. Only when zooming around the origin one can observe a very small shift for the measurements performed with the LBBDS kept on, which can be expressed by very small values in terms of V_OC_ (0.5 mV) and short-circuit current, I_SC,_ (1 nA). In comparison, the I-V curve measured with the system using a wavelength of 690 nm for the LBBDS exhibits a significant photovoltaic effect, with values of V_OC_ and I_SC_ of 545 mV and 28 μA, respectively. This clearly evidences the disruptive effect of a LBBDS with a laser wavelength in the visible range. These results show the difficulties to perform KPFM measurements under real dark conditions when in particular the LBBDS wavelength can interact with the sample. The next illustrated results were all performed with the AFM’s LBBDS operating at 1310 nm described in the Kelvin-Probe subsection.

**Fig. 3:**
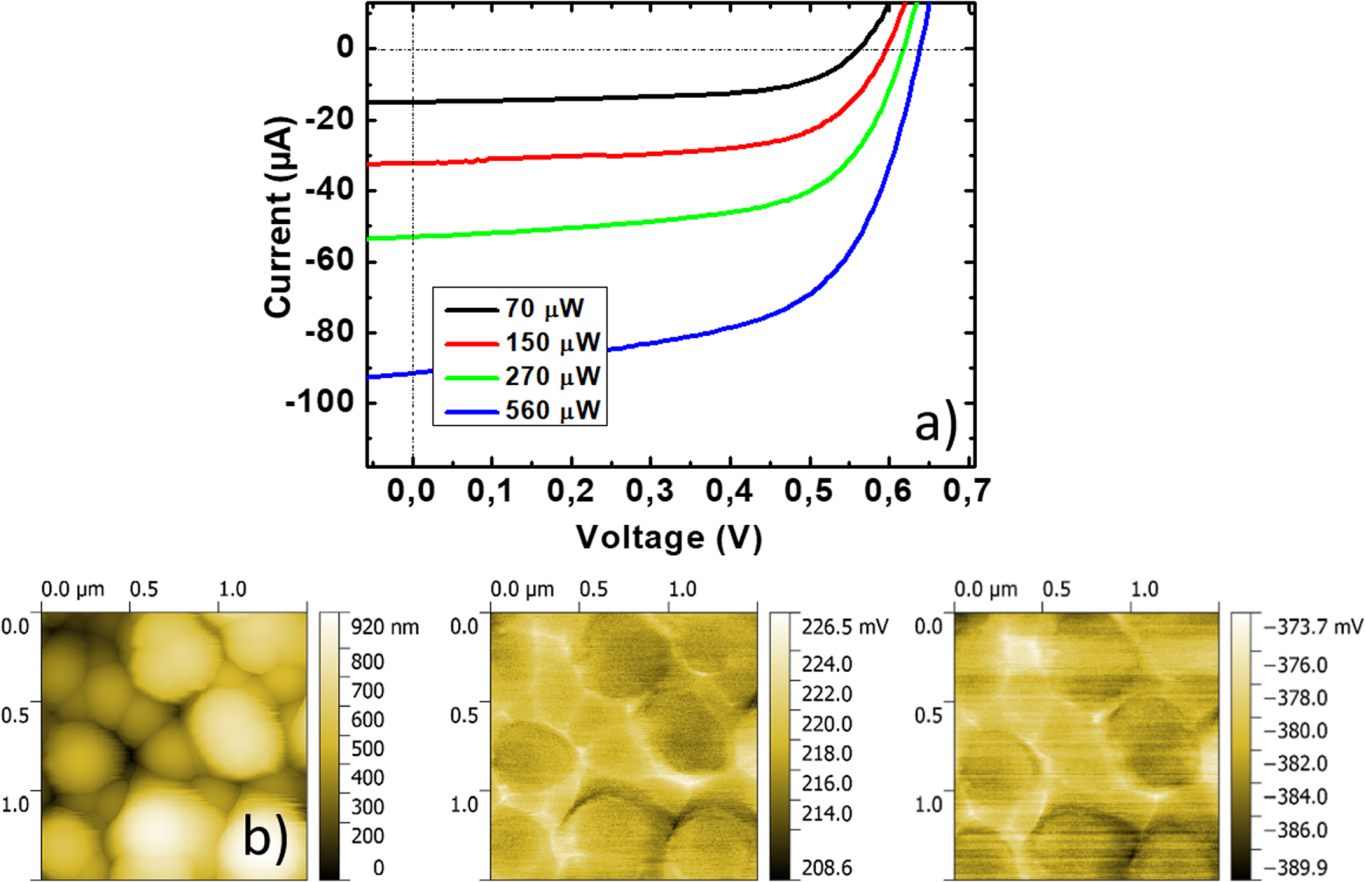
**a** Macroscopic I-V curves measured under different power illuminations (66, 5, 149, 268, and 555 μW at 488 nm); **b** from left to right: topography, CPD under dark conditions, and CPD under illumination (270 μW at 488 nm), respectively

An example of photovoltaic measurement in a completed SiNW RJ device is displayed in Fig. 3. In particular macroscopic I-V measurements under different power illuminations (70, 150, 270 and 560 μW) are presented in Fig. 3.a. The I-V curves show a typical PV cell operating behavior where I_SC_ and V_OC_ increase with the incident light power. Figure 3.b shows an example of KPFM mapping that represents, from left to right, the topography, the CPD under dark and the CPD under 488 nm illumination. The topography scan reveals NWs with heights of several hundreds of nanometers and showing a density per unit area of around 10^9^ cm^-2^. The CPD scans display local potential variations of around ±10 mV taking place mainly at the NW edges. These variations can be considered as artifacts due to the quick change in topography that the AFM tip goes through during the scan motion and in particular when it passes between two NWs. The places which are exempt from such artifact are the top of the NWs where the topography height change remains negligible. All CPD values presented in the following were extracted at the top of the NWs.

**Fig. 4:**
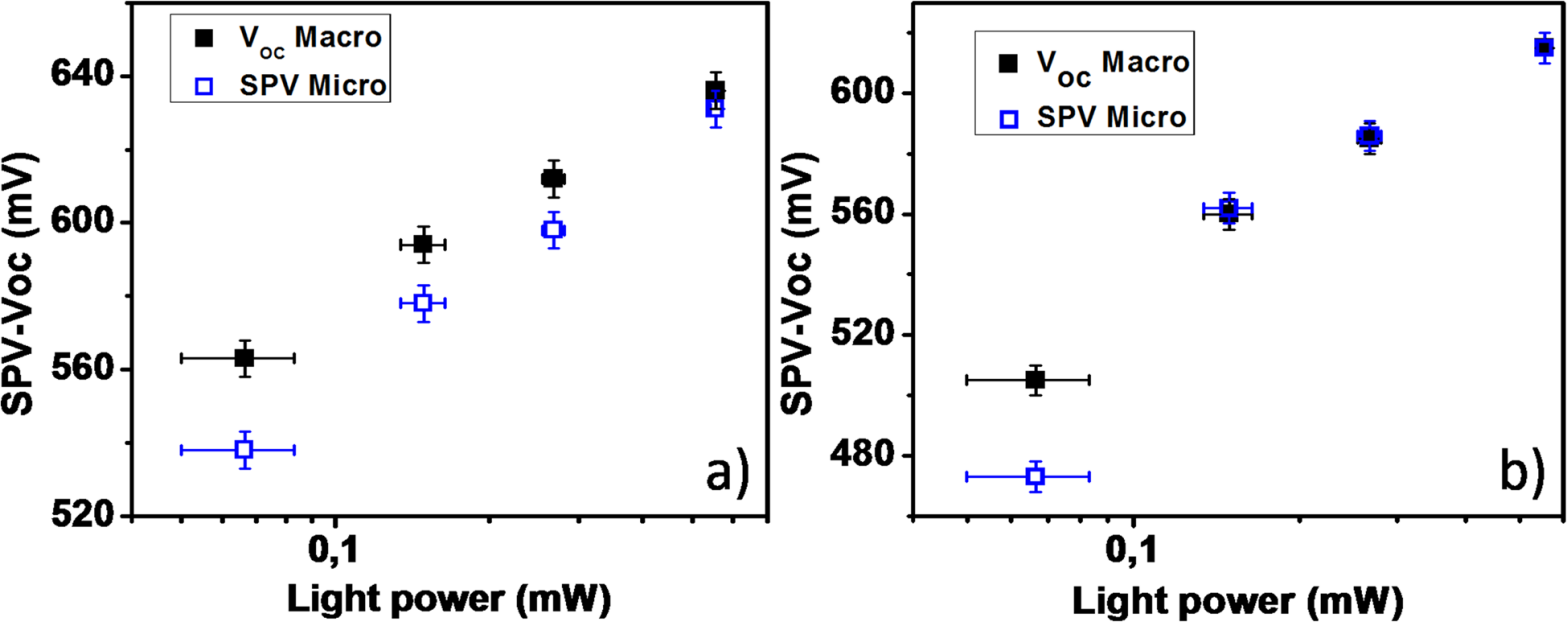
*V*_OC_ and SPV versus light power for two different devices: dev 1 (**a**) and dev 2 (**b**)

**Fig. 5:**
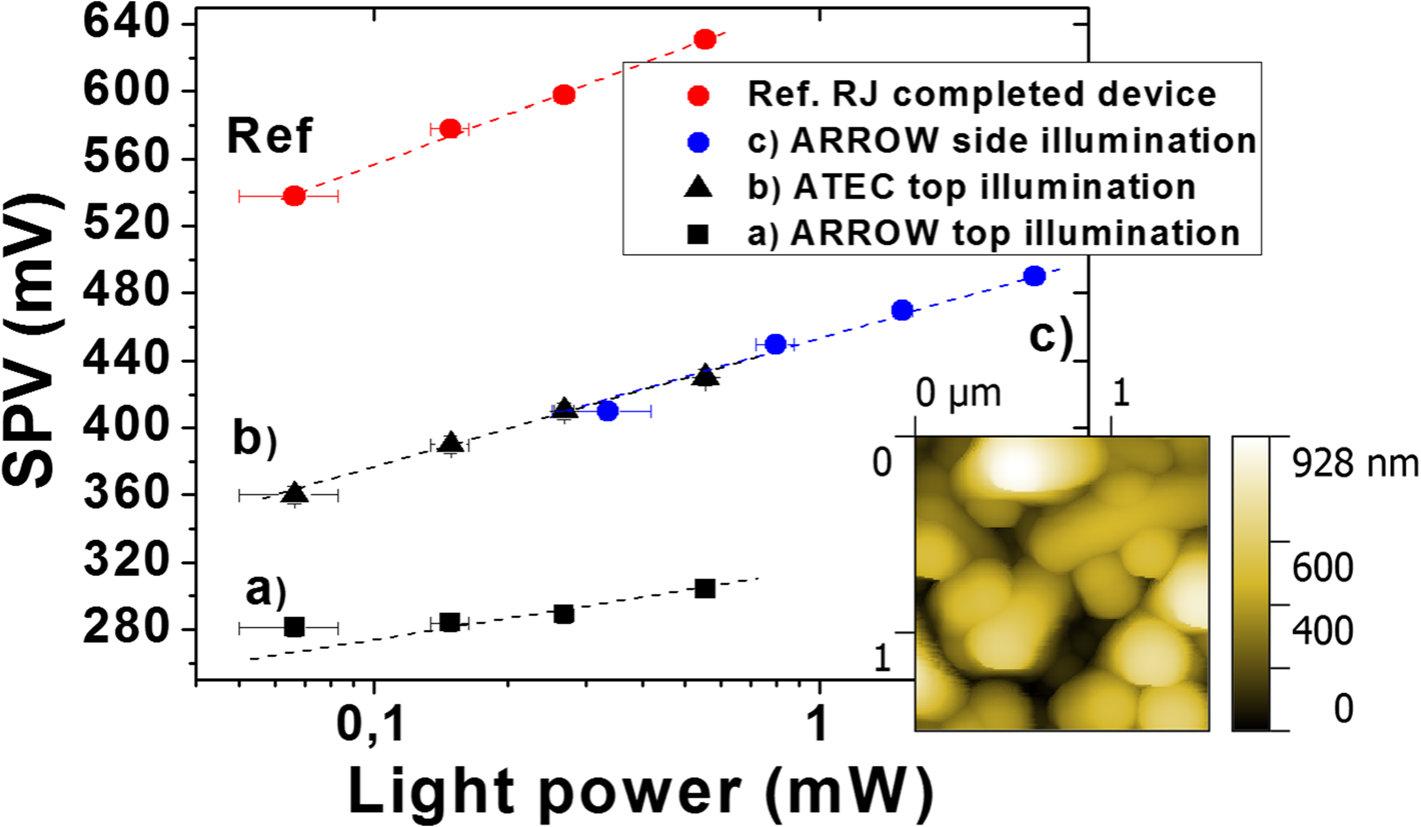
SPV versus light power obtained on isolated RJ NWs. The measurements were performed with different AFM tip shapes (ARROW-EFM and ATEC-EFM) and different directions of illumination (top and side). The reference RJ device designates the device 1 illustrated in Fig. 4a. The AFM image at the bottom right shows an example of the topography measured on isolated NWs

Figure 4 compares the V_OC_ and SPV values extracted from the macroscopic I-V and the KPFM measurements as functions of the incident illumination power. This comparison was performed for two different completed devices and illustrated in a semi-log scale. The maximum difference between the Voc and SPV curves is less than 5% for the lowest illumination power (~70 μW) and becomes less than 2% for higher illumination power. It is important to note that the error bar associated to the experimental evaluation of the impinging light power increases when the illumination power decreases which can explain the difference of 5% between V_OC_ and SPV previously mentioned. For both graphs the SPV and V_OC_ values follow a logarithmic behavior with values in the range 500-600 mV. The slopes of Voc and SPV give an ideality factor (n) of 1.5 ± 0.1 for device 1 and 1.75± 0.25 for device 2, respectively. These values are in good agreement with values reported in the literature for a-Si:H P-I-N junction which are in the range 1.5-2 [12–14]. In Fig. 5 we illustrate measurements of SPV versus light power performed on isolated SINW RJ devices. The term isolated refers here to the fact that the nanowire RJs are not covered with ITO, so they are not electrically connected through the top conductive layer. As a reference guide, the SPV curve obtained previously for the completed RJ device in Fig. 4.a was also shown in Fig. 5. The reported SPV values correspond to an average value resulting from several NWs for scan sizes of 3x3 μm². The SPV measurements on isolated devices were first performed with an arrow shape AFM tip (ARROW-EFM) and an illumination coming from the top just as the SPV measurement performed on the completed device. The very low SPV values for this curve (Fig. 5.a, squares) as well as its slope below 1 (~0.4) suggests a shadowing effect due to the AFM tip. Indeed keeping the same top illumination and changing the AFM tip by a tilted probe (ATEC-EFM) allowed us to observe an increase of 40 % of the SPV values for the same range of power illumination (Figure 5.b, triangle). Similar results were obtained when changing the illumination from the top to the side and replacing the AFM tip ATEC by the initial AFM tip ARROW (Fig. 5.c, blue dots). Although the SPV values have significantly increased compared to the measurements with top illumination and ARROW-EFM tip, they remain below the reference one while keeping similar slopes (~1.3-1.4). Note that this shadowing effect was not observable in case of completed devices because for this this configuration, the SPV images the photovoltage of the entire device: thousands of nanowires connected together by the ITO front contact.

**Fig. 6:**
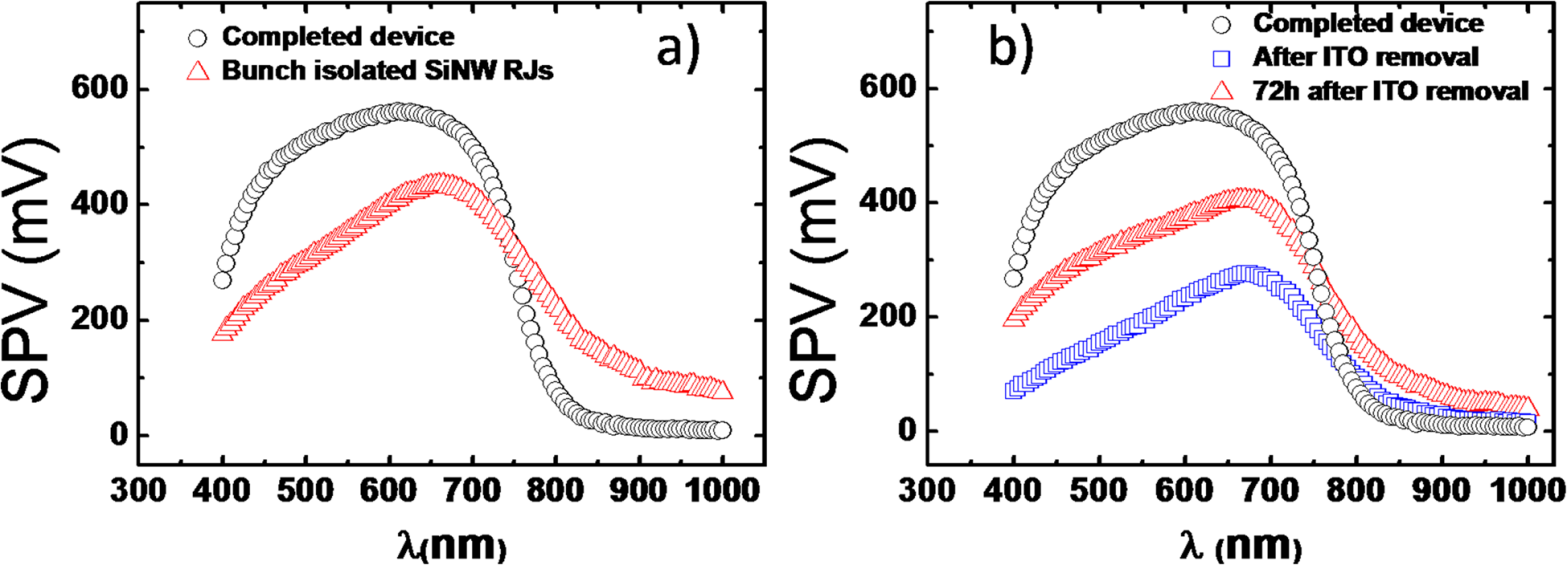
SPS measurements performed on **a** a completed device and a bunch of isolated SiNWs; **b** a completed device, just after removing ITO, and 72 h later

To complement those results, qualitative SPS analysis was performed above a bunch of isolated devices and then above a completed device. Fig. 6.a displays the obtained SPV spectra with clear differences across the entire spectrum. It is interesting to underline that the completed device shows a negligible SPV (~10 mV) in the near infrared (NIR) region with a SPV threshold taking place around 800 nm and below which the SPV increases rapidly reaching a maximum of 560 mV at 630 nm. Conversely, the bunch of isolated devices reveals a significant SPV of 80-260 mV in the NIR (800-1000 nm) that increases gradually with decreasing wavelength, up to 435 mV for 665 nm. Below 665 nm and 630 nm both SPV curves decrease with decreasing wavelength which may be linked to the expected decrease of the irradiance of the halogen lamp used in this setup (as mentioned above, the SPS approach here is based on qualitative measurements since the flux cannot be kept constant). In a second approach SPS measurements were performed on a completed device and after locally removing the ITO top contact with 1% HF solution applied as a drop on the device. Figure 6.b illustrates these measurements, and the SPV spectra were specifically collected just after removing ITO and 72 hours later. The removal of the ITO layer has a major effect on the SPV spectrum when compared to the completed device. A strong decrease of the SPV signal is observed in the range 400-750 nm just after the ITO removal. After 72 hours the SPV signal stabilizes at a higher level which can differs, depending on the wavelength, by more than a factor of 2. It also turns out that the SPV signal slightly increases at longer wavelengths ( λ>750 nm). Comparing the SPV spectra of Fig. 6, it appears that after the ITO removal illustrated in Figure 6.b and especially after 72 h stabilization the NW devices show a similar state than those designated as bunch of isolated NWs in Fig. 6.a, the latter having never had any ITO coating. Another important observation concerns the SPV signal measured at 488 nm which value is a factor ~1.7 lower for a bunch of isolated NWs than for a completed device. This observation supports the SPV results of Fig. 5 performed by KPFM on isolated NW RJs with an illumination at 488 nm. Indeed, despite the optimization of the AFM tip shape and the illumination conditions, the measured SPV values were also lower than that of the completed device by a factor varying between 1.5 and 2, depending on the illumination power.

The results of Fig. 6 clearly show that the ITO top contact is required to develop higher values of SPV (i.e. V_OC_) and more specifically the key point remains the interface (n) a‑Si:H/ITO. This interface is characterized by a very thin n-type a-Si:H layer (~ 10 nm) in order to favor the optical transmission. The doping level of this layer and the ITO work function can in particular cause the full depletion of the a-Si:H layer. Thus, a sudden drop of potential can take place across the interface before reaching a flat band potential in the ITO. Such a drop in potential at the interface with the ITO top contact has already been illustrated in P‑I‑N a-Si:H structures that were analyzed by SPV profiling [12, 15]. Same interfaces with ultrathin a‑Si:H layers were also investigated in the solar cell technology of a‑Si:H/crystalline Si heterojunction emphasizing again the impact of the doping level and the thickness of the a-Si:H layer on the V_OC_ with and without ITO [16, 17].

The previous considerations indicate that the local SPV analysis by KPFM on isolated NW RJs cannot quantitatively reflect the optimal value of V_OC_ due to the absence of ITO. The extracted local V_OC_ is here restricted by the surface band bending as a consequence of the full depletion of the n-type a-Si:H layer and its oxidation surface state. The measured SPV not only includes the V_OC_ but also the photo induced band-bending change near the surface of the n-type a-Si:H layer [18].

## Conclusion

Completed devices based on RJ SiNWs were jointly analyzed under illumination by I-V and KPFM measurements. This first comparison carried out for different illumination powers shows that the local SPV values extracted from KPFM are very close to the V_OC_ values obtained from I-V analysis. Local SPV measurements on isolated RJ SiNWs show, on the contrary, a significant difference from the previous V_OC_ values. A shadowing effect of the AFM tip has been evidenced and minimized changing the tip shape and/or the illumination orientation. The optimized SPV values gathered from isolated RJ SiNWs show a logarithmic behavior with the illumination power but remain well below the V_OC_ reference values. SPS analysis performed on bunches of isolated SiNW devices highlight the absence of the interface (n) a-Si:H /ITO as the cause of the loss of potential, and notably because the studied isolated SiNW devices do not have ITO as top contact. Despite this, the local SPV extracted on isolated SiNW devices under different illumination conditions shows a linear correspondence with the V_OC_ measured on completed devices, confirming in particular that local SPV can mirror the V_OC_.

## Data Availability

The datasets used and/or analyzed during the current study are available from the corresponding author on reasonable request.

## Abbreviations

AFM: Atomic force microscopy
AM: Amplitude modulation
a-Si:H: Hydrogenated amorphous silicon
Cg: Corning glass
CPD: Contact potential difference
FM: Frequency modulation
ITO: Indium-tin-oxide
I-V: Current-voltage
KPFM: Kelvin probe force microscopy
LBBDS: Laser beam-based deflection system
n: Ideality factor
NW: Nanowire
PECVD: Plasma-enhanced chemical vapor deposition
PV: Photovoltaic
RJ: Radial junction
SiNW: Silicon nanowire
SPS: Surface photovoltage spectroscopy
SPV: Surface photovoltage
*V*_OC_: Open circuit voltage
